# Learning to rank diversified results for biomedical information retrieval from multiple features

**DOI:** 10.1186/1475-925X-13-S2-S3

**Published:** 2014-12-11

**Authors:** Jiajin Wu, Jimmy Xiangji Huang, Zheng Ye

**Affiliations:** 1 Information Retrieval and Knowledge Management Research Lab, York University, 4700 Keele Street, M3J1P3 Toronto, Canada; 2 Information Retrieval and Knowledge Management Research Lab, School of Information Technology, York University, 4700 Keele Street, M3J1P3 Toronto, Canada

## Abstract

**Background:**

Different from traditional information retrieval (IR), promoting diversity in IR takes consideration of relationship between documents in order to promote novelty and reduce redundancy thus to provide diversified results to satisfy various user intents. Diversity IR in biomedical domain is especially important as biologists sometimes want diversified results pertinent to their query.

**Methods:**

A combined learning-to-rank (LTR) framework is learned through a general ranking model (gLTR) and a diversity-biased model. The former is learned from general ranking features by a conventional learning-to-rank approach; the latter is constructed with diversity-indicating features added, which are extracted based on the retrieved passages' topics detected using Wikipedia and ranking order produced by the general learning-to-rank model; final ranking results are given by combination of both models.

**Results:**

Compared with baselines BM25 and DirKL on 2006 and 2007 collections, the gLTR has 0.2292 (+16.23% and +44.1% improvement over BM25 and DirKL respectively) and 0.1873 (+15.78% and +39.0% improvement over BM25 and DirKL respectively) in terms of aspect level of mean average precision (Aspect MAP). The LTR method outperforms gLTR on 2006 and 2007 collections with 4.7% and 2.4% improvement in terms of Aspect MAP.

**Conclusions:**

The learning-to-rank method is an efficient way for biomedical information retrieval and the diversity-biased features are beneficial for promoting diversity in ranking results.

## Background

How to promote diversity in ranking for information retrieval has become a very hot topic [1-7] in the past decade. One of the major reasons is the increasing demand of novelty and disambiguation of user query, as described in [8] as Intrinsic Diversity and Extrinsic Diversity respectively. Beyond counting on relevance between documents and query, diversity IR takes consideration of relationship among documents in ranking order to promote diversity and reduce redundancy. In essence, to promote diversity means to provide various aspects of information in the ranking results list and to reduce redundancy aims to deduce repeatedly mentioned information.

The application of diversity IR has drawn great attention and shown beneficial in previous studies when query turns out to be ambiguous, especially in the scenario of biomedical IR investigated in TREC 2006 and 2007 Genomics tracks where biologists tend to query a certain type of entities covering different aspects that are related to the question, for example, genes, proteins, diseases, and mutations.

In the TREC 2006 Genomics track, University of Wisconsin re-ranked the passages using a clustering-based approach named GRASSHOPPER to promote ranking diversity [9]. GRASSHOPPER is an alternative to MMR [1] and variants with a principled mathematical model and strong empirical performance on artificial data set [10]. Later in the 2007 track, most teams tried to obtain the aspect level performance through their passage level results, instead of working on the aspect level retrieval directly [11-13].

Recent works [14,4] show that Wikipedia can be used as an external knowledge resource to facilitate biomedical IR. In these studies, Wikipedia is used as an encyclopaedia to help to detect the topics of documents. The novelty of detected topics are measured by binary novelty measurement [4] or survival models [14] for re-ranking to promote diversity of whole ranking list.

Besides methods mentioned above, recently there are some papers dealing with diversity IR using learning-to-rank methods. One typical work is to directly learn a diversified ranking of documents based on users' clicking behavior, and the algorithm maximizes the probability that a relevant document is found in the top k positions of a ranking [15]. Another work is to optimize variants of traditional IR metrics, such as NDCG and ERR, in the way of rewarding aspect coverage thus to penalize redundancy [16]. However, during the model learning process of these methods, only general features are used while none diversity-related features are considered. To the best of our knowledge, there is no learning-to-rank algorithm that addresses the specific features that may reflect the novelty of single document and the diversity of whole ranking list. We believe that with this general representation, the benefits brought by learning-to-rank may not have been fully exploited as the novelty and diversity characteristics of ranking lists are ignored. We argue that it is promising to define and make use of diversity reflecting features to better model diversity information.

In this paper, we propose several features that capture diversity of documents and construct a combined learning-to-rank framework (LTR) by integrating a general ranking model with the diversity-biased model. Our approach adopts the idea of measuring the topics' novelty of documents together with diversity of ranking list. We find a way to combine this dynamic changing feature with the learning-to-rank technology. In our proposed framework, firstly the general ranking model is learned from general ranking features by a conventional learning-to-rank approach; secondly diversity features are extracted based on the retrieved passages' topics detected using Wikipedia and ranking order produced by the general learning-to-rank model; then, a diversity-biased ranking model is constructed from diversity-indicating features together with conventional features; final ranking results are given by combination of both models.

The major contributions of this paper are two-fold. First, we propose several diversity-reflecting features by studying the relationship among documents. Second, we propose a learning-to-rank framework to combine the diversity-biased model with a general ranking model learned from the common features. Extensive experiments on the TREC 2006 and 2007 Genomics tracks [12,17] demonstrate the effectiveness of our proposed diversity-favored learning-to-rank approach.

## Methods

We propose a learning-to-rank framework that utilizes both the common features of biomedical text, and the diversity information, specifically novelty and freshness of retrieved results in terms of topics and coverage of different query aspects, which can be expressed and obtained in many ways, for example, using topic model and clustering. The proposed framework consists of a general ranking model and a diversity-biased ranking model. More specifically, the general ranking model is learned from the training instances represented by the traditional learning-to-rank features common to ad-hoc IR tasks. The diversity-biased model is learned from both general features and diversity-biased features proposed in this paper. The final learning-to-rank model (LTR) is combined linearly as follows:

(1) LTR(d,Q)=α⋅gLTR(d,Q)+β⋅dLTR(d,Q)

where *gLTR*(*d, Q*) is the general learning-to-rank model and *dLT R*(*d, Q*) the diversity-biased model, and *α *and *β *are parameters that control the weight of two parts and they have the relationship of *β *= 1 − *α*.

To deploy our proposed learning-to-rank framework in practice, firstly a general ranking model is learned from a set of training queries with their associated relevance assessments information. Next for the first pass retrieval results obtained from the general ranking model, we use Wikipedia Miner to extract their related topics. From this ranking list and the topics information, we generate the diversity-biased features for each query-passage pair. Then the diversity-biased learning-to-rank model is learned based on all these features.

### General learning to rank model

#### General features extraction

Learning-to-rank has shown advantage in incorporating various evidences to design a unified ranking model for enhancing IR [18]. Typical features being utilized for constructing an learning-to-rank model include content-based and non contend-based (e.g. linkage features). In this paper, due to the data being scientific publications, we choose the content-based features extracted from each query-passage entries as shown in Table 1.

**Table 1:** Features for general learning-to-rank model.

Feature	Description
**TF-IDF**	Term frequency inverse document frequency.

**BM25**	Okapi BM25 model [21].

**DFR BM25**	The DFR version of BM25 [23].

**InL2**	An algorithm derived from the divergence from randomness (DFR) framework [23].

**DLH13**	An DLH hyper-geometric DFR model (parameter free) [23].

**DirKL**	KL-divergence language model with Dirichlet smoothing [22].

**Hiemstra LM**	Hiemstra's language model [24].

**ProxQT**	Proximity of Query Terms: Intuitively, the more close the query terms occur in a document, the more likely the document would be relevant [25].

It can be seen that our general features contain different paradigms of state-of-the-art IR models, which are usually used as strong baselines in previous studies.

#### Learning to rank algorithm

Many learning-to-rank approaches have been proposed in the literature [18], which can be applied for learning the general ranking model. Among many of these approaches, we choose to use the coordinate ascent algorithm proposed in [19], which directly optimizes the parameters in the interest of maximizing retrieval metric and has been proven to be highly effective for a small number of parameters [20], and has good empirically verified generalization properties. It could be achieved by solving the following statement:

(2) $$\begin{array}{c} \hat{\Lambda }=\text{arg}\underset{\Lambda }{\text{max}}E\left(R_{\Lambda };T\right)\\ s.t.\;R_{\Lambda }\sim S_{\Lambda }\left(D;Q\right)\\ \;\;\;\Lambda \in M_{\Lambda } \end{array}$$

where *S*_Λ_(*D*; *Q*) is a scoring function parameterized by a vector of parameters Λ, and it is computed for each query *Q *with each document *D *in documents set D(D∈D), $E\left(R_{\text{Λ}};T\right)$ is an evaluation matrix, $R_{\text{Λ}}\sim S_{\text{Λ}}\left(D;Q\right)$ denotes that the orderings in $R_{\text{Λ}}$ are induced using scoring function S, and *M*_Λ _is the parameter space over Λ.

### Diversity-biased learning to rank

#### Diversity features

We consider the task of promoting diversity as such a scenario that a user would prefer a ranking list of passages so that the top returned passages should be as relevant as possible and meanwhile the passages should cover as many different aspects as possible. Therefore when generating the ranking list, the aspects difference between passages should be taken into consideration to ensure good coverage of different aspects and low redundancy. In such a direction, we propose the diversity-biased features as shown in Table 2.

**Table 2:** Additional features for diversity-biased learning-to-rank model.

Feature	Description
**#RelAsp**	Number of relevant aspects the passage contains.

**#NonRelAsp**	Number of irrelevant aspects the passage contains.

**#NewRelAsp**	Number of new relevant aspects the passage contains compared with afore ranked passages.

**#OldRelAsp**	Number of relevant aspects that already existed in afore ranked passages.

**NewAspPsg**	Ratio of passages that contains new aspects with all afore ranked passages.

**%RelAsp**	Ratio of number of relevant aspects with all aspects before current rank position.

**%UniqRelAsp**	Ratio of unique relevant aspects with all aspects before current rank position.

#### Features extraction and model strategy

Our assumption is that there is a perfect diversified ranking list and through learning from the general features, which represent the value of each individual query-passage pair, and diversified features, which capture the novelty and diversity of the whole ranking list, we can get an oracle ranking model for further directing ranking for new dataset.

As can be seen in the previous section, the diversity features aim to reflect the relationship between current document with former ranked documents and therefore the features extraction is related to certain documents ranking and their quality are potentially affected by the ranking list. Actually this simulates the process of generating diversified documents based on former ranked documents in the paradigm of re-ranking for promoting novelty and diversity, where the document for each position is determined in the principle of maximizing the diversity for the whole ranking list. Accordingly these diversity features should be extracted in tandem. There can be different ways to generate diversity features:

• **Once for all**: The diversity features are generated according to the initial ranking given by general learning-to-rank model, and the oracle model is learned from all features once for all.

• **Dynamic update**: After the diversity features of documents in *ith *top *K *subset are determined, the oracle learning-to-rank model will be re-learned and consequently the general ranking will be updated which results in the re-generating of diversity features.

Heuristically the second strategy might be better; however, we argue that this is much time-consuming and complicated in practice. Therefore in this paper we adopt the first strategy for diversity feature generation.

## Results

### Experimental settings

In order to evaluate the proposed approach, we use the TREC 2006 and 2007 Genomics tracks full-text collection as the test corpus, which consists of 162,259 documents from 49 genomic-related journals indexed by MEDLINE [17,12] including 64 queries in total. Three levels of retrieval metrics were measured in the TREC 2006, namely Passage MAP, Aspect MAP, Document MAP and one more were proposed in TREC 2007 Genomics track, i.e., Passage2 MAP [17,12].

Golden standard of relevance and aspects judgment for official released legal span of passages are provided. For the sake of generalization, we only utilize the relevance information for generalizing train file for general learning-to-rank model. We define passage as maximum span of consecutive text within one single document not including any HTML paragraph tag. Within this principle we extract passages from the meta data and index. In constructing the train dataset for learning-to-rank, we compare the extracted passages with the official defined passages with golden standard of relevance and assume whenever there is an overlap, the relevance of official released passages will contribute to extracted passage.

Parameters of learning-to-rank algorithm is optimized using a greedy boosting method on 2-fold cross-validation setting in which the best model is selected according to Document MAP. The parameters *α *and *β *in Equation 1 are tuned based on 2-fold cross-validation.

### Results and analyses

#### Comparison with baseline

We use BM25 [21], Language Model [22] (DirKL) and state-of-the-art learning-to-rank model [19] (gLTR) as strong baselines in the experiments. The comparison of the proposed method (LTR) with the baselines are presented in Table 3 and Table 4. The "+" sign and number in parentheses indicate the statistical significant improvements of LTR over gLTR using Student's t-test at alpha level of 0.05. Bold font denotes the best performance on certain metric of the four methods.

**Table 3:** Performance Comparison with Baselines on 2006 Collection.

MAP	Aspect	Passage	Document
BM25	0.1972	0.0362	0.3449
DirKL	0.1591	0.0360	0.3566
gLTR	0.2292	0.0369	0.3547
LTR	**0.2400** (+4.7%)	**0.0416** (+12.7%)	**0.3910** (+10.23%)

**Table 4:** Performance Comparison with Baselines on 2007 Collection.

MAP	Aspect	Passage	Passage2	Document
BM25	0.1622	0.0651	0.0697	0.2402
DirKL	0.1383	0.0693	0.0637	0.2376
gLTR	0.1878	0.0533	0.0706	0.2179
LTR	**0.1923** (+2.4%)	**0.0784** (+47.1%)	**0.0831** (+17.7%)	**0.2721** (+24.9%)

As can be seen from Table 3 and Table 4 when diversity features are utilized for learning a ranking model, performance improvements over three strong baselines BM25, DirKL and gLTR can be obtained in terms of all different levels of MAP metrics on both 2006 and 2007 Genomics Track tasks. As to the higher improvement space of Passage MAP than Aspect MAP, we attribute it to the paragraph-based indexing of the original data and the way how we generate training dataset for learning-to-rank: the relevance of passages are contributed by all embedded paragraphs that are relevant while referring to different topics of the query.

It is noticeable that the improvements of Document MAP are also remarkable. This shows that the diversity features are beneficial for promoting not only diversity but also general relevance performance. When the diversity information is used for training model, the passages that are both relevant and have various topics will be favored by the ranking model. This is promising in that when being designed properly, the diversity features are beneficial both in improving general IR metrics and promoting diversity in ranking.

#### Comparison with TREC results

We also compare our results with the TREC submission results in Table 5 and Table 6. The italic bold font in Table 5 and Table 6 denotes the second best result in each matrix. Normally it is not fair to compare with the best TREC result because the submission could comprehensively use many resources, but the median result shows the average level of all submissions. So the outperforming median results at least shows our model is promising.

**Table 5:** Performance Comparison with TREC 2006 Submissions.

MAP	Aspect	Passage	Document
Max	**0.4411**	**0.1486**	**0.5439**
Min	0.011	0.0007	0.0198
Median	0.1581	0.0345	0.3083
gLTR	0.2292	0.0369	0.3547
LTR	***0.2400***	***0.0416***	***0.3910***

**Table 6:** Performance Comparison with TREC 2007 Submissions.

MAP	Aspect	Passage	Passage2	Document
Max	**0.2631**	**0.0976**	**0.1148**	**0.3286**
Min	0.0197	0.0029	0.0008	0.0329
Median	0.1311	0.0565	0.0377	0.1897
gLTR	0.1878	0.0533	0.0706	0.2179
LTR	***0.1923***	***0.0784***	***0.0831***	***0.2721***

#### Comparison with re-ranking method

Yin et al [4] proposed a cost-function re-ranking method based on detected aspects using Wikipedia for promoting diversity in biomedical IR. The re-ranking tactic can be deployed on the basis of arbitrary ranking result. For example, re-ranking on 2007 collection on top of that year's best result receives further improvement. Therefore we compare our performance of combined ranking model with re-ranking method results in Table 7 and Table 8. No statistical test is conducted due to lack of their result files. Bold font denotes the best result.

**Table 7:** Comparison with Re-Ranking Method on 2006 Collection.

MAP	Aspect	Passage	Document
Re-Rank	0.2374	0.0386	0.3549
LTR	**0.2400**	**0.0416**	**0.3910**

**Table 8:** Comparison with Re-Ranking Method on 2007 Collection.

MAP	Aspect	Passage	Passage2	Document
Re-Rank	0.1642	0.0651	0.0679	0.2116
LTR	**0.1923**	**0.0784**	**0.0831**	**0.2721**

As shown in Table 7 and Table 8 our method achieves performance improvements over the re-ranking method in terms of all metrics on both 2006 and 2007 collections. We attribute this to the diversity-representative features proposed in this paper and the utilization of learning-to-rank technology. Learning-to-rank has demonstrated strength in integrating multiple sources of features in constructing model. As well as other machine learning methods, features play an important role in learning-to-rank. As proven usefulness in the previous section, diversity-representative features essentially enhance the learning-to-rank method with greater opportunity to capture novelty and diversity information in ranking list which results in building better ranking model.

In summary, from the results and analyses we can draw a conclusion that our proposed diversity features are representative of diversity information of ranking list and helpful in advancing ranking model within the combined learning-to-rank framework proposed in this paper.

#### Effect of control parameter

In this section, we evaluate the parameters *α *and *β *in the framework that can affect the retrieval performance. Because *β *= 1 *− α*, so in this section, we present the results under different settings of *α*, more specifically we sweep over values (0.1, 0.2, ..., 0.9).

In particular, for each dataset we conduct a 2-fold cross-validation, where each fold randomly chooses half of the topics for training and the remaining for testing, and vice versa. The overall retrieval performance is averaged over the two test topic sets.

**Figure 1:**
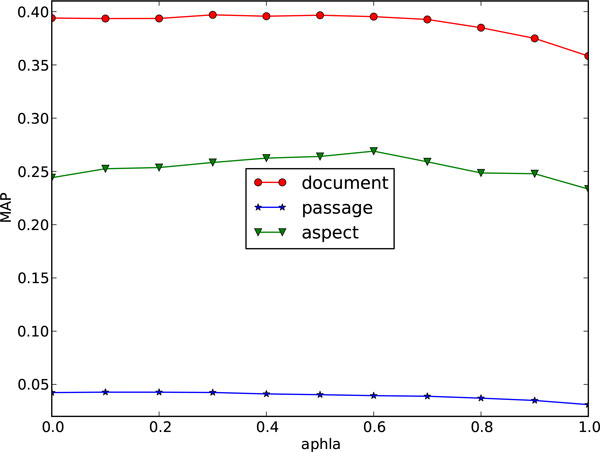
**Parameter *α *against the retrieval performance on 2006 collection**.

**Figure 2:**
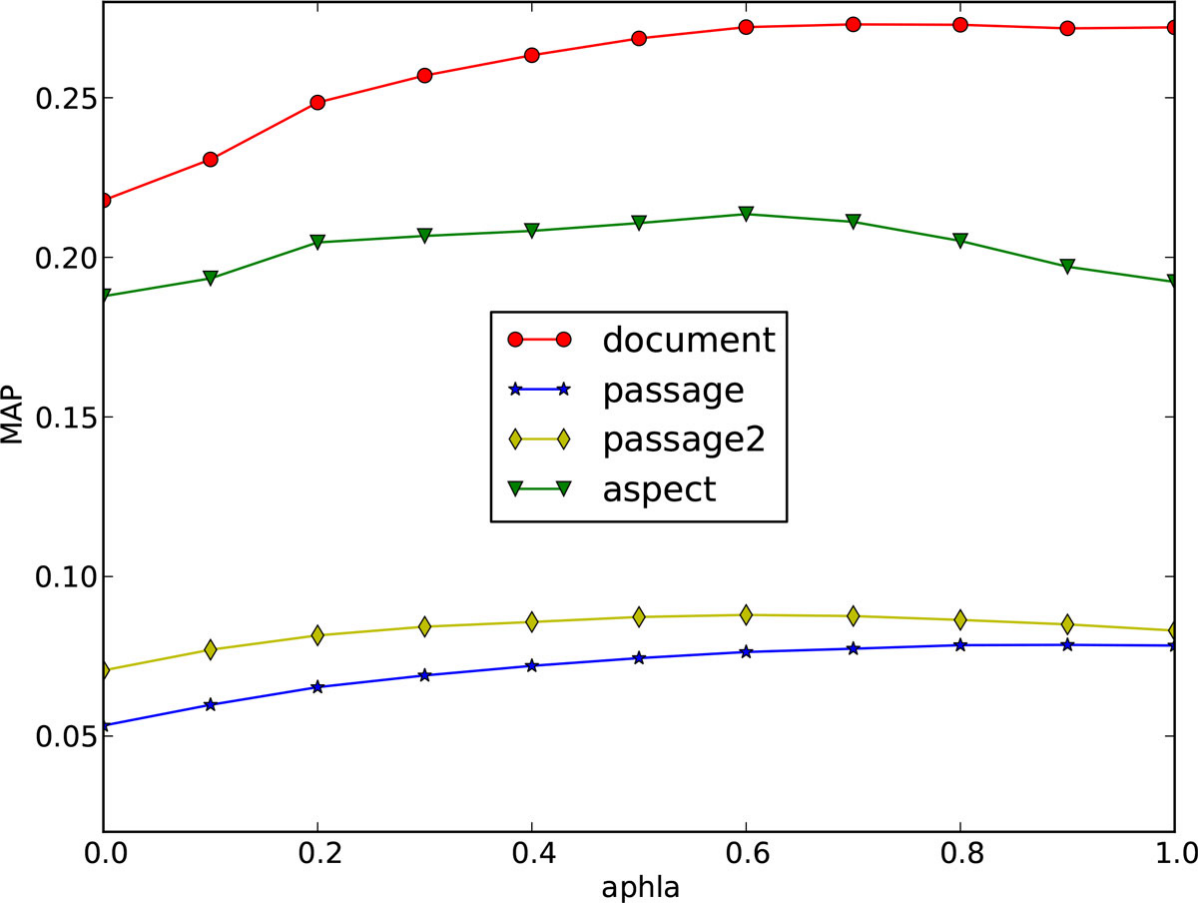
**Parameter *α *against the retrieval performance on 2007 collection**.

It can be known from Figure 1 and Figure 2 that the retrieval performance on both 2006 and 2007 data collections are relatively stable under different settings of parameter *α*, which has significance in practice because the combined model will not be largely affected by different parameter settings and could be free from parameter tuning.

It is also noticed that when *α *is set to 1, the combined model in Equation 1 is equal to gLTR, which is the general model, while it is set to 0, the combined model equals to the diversity-biased model, and neither of them obtain the best result. This shows the necessity and effectiveness of the combination. For some matrices (eg. document MAP on 2007 collection and aspect MAP on both collections), the best result occurs when *α *is set in the range of (0.6, 0.8). So the empirical setting of parameter *α *is suggested to be (0.6 ~ 0.8) when no training data is available.

## Conclusions

In this paper, we have applied learning-to-rank technology to biomedical information retrieval and proposed a combined learning-to-rank model which integrates a general ranking model and a diversity-biased model. The general ranking model proved to be effective. However, with the help of diversity-biased model, the retrieval results are more promising. The diversity-biased model is learned from both general features and diversity-favored features to award ranking list with low redundancy and high diversity. The diversity-reflecting features which are defined in the perspective of topics relationship of different passages in ranking order appear to contribute promoting results diversity.

## Competing interests

The authors declare that they have no competing interests.

## Authors' contributions

J Wu conceptualized the project. J Huang approved the project and collected the data. J Wu and Z Ye conducted the experiments. J Wu wrote the drafted manuscript. J Huang and Z Ye critically reviewed and revised many versions of the drafted manuscript. All of the authors read and approved the manuscript.

## Declarations

Publication of this article has been funded by the Natural Sciences and Engineering Research Council (NSERC) of Canada, the Early Researcher Award/Premier's Research Excellence Award and the IBM Shared University Research (SUR) Award. We also would like to thank IBM Canada for providing IBM BladeCenter blade servers to conduct experiments reported in the paper.

This article has been published as part of *BioMedical Engineering OnLine *Volume 13 Supplement 2, 2014: Selected articles from the IEEE International Conference on Bioinformatics and Biomedicine (BIBM 2013): BioMedical Engineering OnLine. The full contents of the supplement are available online at http://www.biomedical-engineering-online.com/supplements/13/S2.
